# Supplementary material for: Uniform spatial pooling explains topographic organization and deviation from receptive-field scale invariance in primate V1
Source: Nat Commun. 2020 Dec 15;11:6390. doi: 10.1038/s41467-020-19954-9 (PMC7738493; doi:10.1038/s41467-020-19954-9)
Supplement: Supplementary file 1 — Supplementary Information [file 41467_2020_19954_MOESM1_ESM.pdf]

Supplementary Information

I. Maps of spectral energy and retinotopy in macaque V1, using GCaMP6f

Recent studies using the synthetic calcium indicator OGB identified maps of preferred spatial frequency (SF) and their relationship to some other established maps in macaque V1 (Nauhaus et al., 2012a, 2016). Here, we replicate some of these results, but with the calcium indicator GCaMP6f. Different calcium indicators are known to have distinct dynamics, contrast between neuropil and cell-bodies, and nonlinearity.

We first examined the micro-organization of orientation and preferred SF (f_o). Visual inspection of the orientation (Fig. S1a,b) and f_o color maps (Fig. S1d,e) reveals a clustering of tuning preference. Clustering was quantified by computing the Pearson correlation of the tuning preference between neuronal pairs (DeAngelis et al., 1999), as a function of cortical distance between the pairs (Nauhaus et al., 2012a). Orientation is a circular parameter, which precludes the use of standard correlation coefficients, so we used a normalized measure of "directional similarity" (Nauhaus et al., 2012a), along with random shuffling to assess significance (Methods). The metric yields +1 if the pairs all have the same orientation and -1 if they are all 90° apart. It yields 0 if the pairs are uncorrelated or tuned 45° apart. For f_o, we used the Pearson correlation between the log of the pair of f_o's. As shown by the first two data points in Figs. S1c,f, when two neurons in an ROI are close (< 150 μm), both f_o and orientation preference are significantly correlated. At greater distances (~300 μm separation), they become significantly negatively correlated. Together, this replicates the previous finding with OGB that orientation and f_o maps are periodic, with a similar spatial period. We also replicated the result that the joint pairwise relationship between f_o and orientation preference is anticorrelated. We computed the pairwise correlation coefficients between the absolute change in f_o and the absolute change in orientation preference. At the distance bin between 150 and 225 μm, the correlation was -0.115 (p = 0.008). The joint correlation was not significant at the other distance bins.

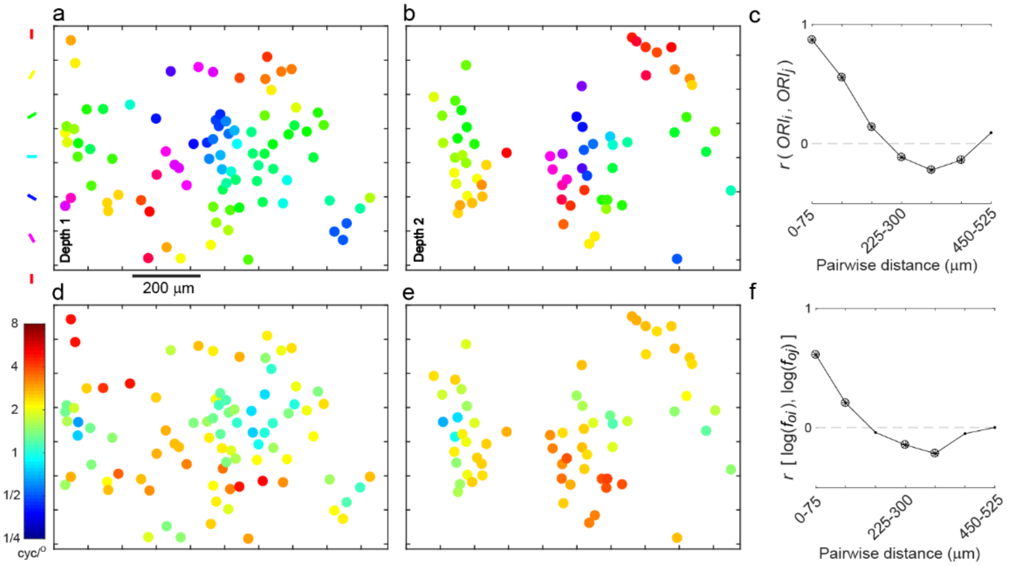

Figure S1: Maps of orientation and f_o. (a,b) Maps of orientation preference at two different depths in layer 2/3, and same lateral position. (c) Pairwise correlation of orientation preference (see Methods for metric used for circular variables) computed for all 3 ROIs in the data set, binned by cortical distance. Each bin has edges that are 75 μm apart. The symbol at each distance bin indicates the correlation's level of significance: A small dot is p>0.01, asterisk is p<0.01, asterisk and open circle is p<0.001. (d,e) f_o maps from the same population as in 'a' and 'b'. (f) Pairwise Pearson correlation coefficient of $\log_2(f_o)$, binned by cortical distance between pairs. Same population of cell pairs as in 'c'. Like in 'c', the symbol indicates the p-value.

Next, we characterized the micro-organization of RF tiling using responses to the random bar stimulus. Fig. S2 shows maps of orientation preference and retinotopy from the same ROIs as in Figure S1. Also shown are the population of orientation tuning curves and RF envelopes, which illustrates the

diversity of orientation tuning and contrasting redundancy of RF spatial coverage. Although the maps of RF position exhibit a retinotopic gradient, the RF envelopes encode overlapping regions of visual space. The RF scatter, computed as the mean distance (°) of the RFs' centers from an x/y planar fit (°), divided by the average RF width (1σ), was 0.2, 0.25, and 0.34 in the 3 ROIs from this study. Although these values are about 10% higher than previously found with OGB (Nauhaus et al., 2016), they confirm the general finding that retinotopy is quite smooth in L2/3 of macaque V1. The difference could be due to OGB vs. GCaMP6 nonlinearities. The GCaMP6 nonlinearity is more "accelerating" than "saturating" (Akerboom et al., 2012; Chen et al., 2013), making it more likely to yield smaller RFs than OGB, thus increasing the computed scatter. Section V below goes into greater depth on the impact of fluorescence nonlinearities on main results. In either case, it is worth noting that RF "outliers" have never been found – e.g. a neuron with a RF center that is displaced from the planar fit of retinotopy by more than 1σ of its RF size.

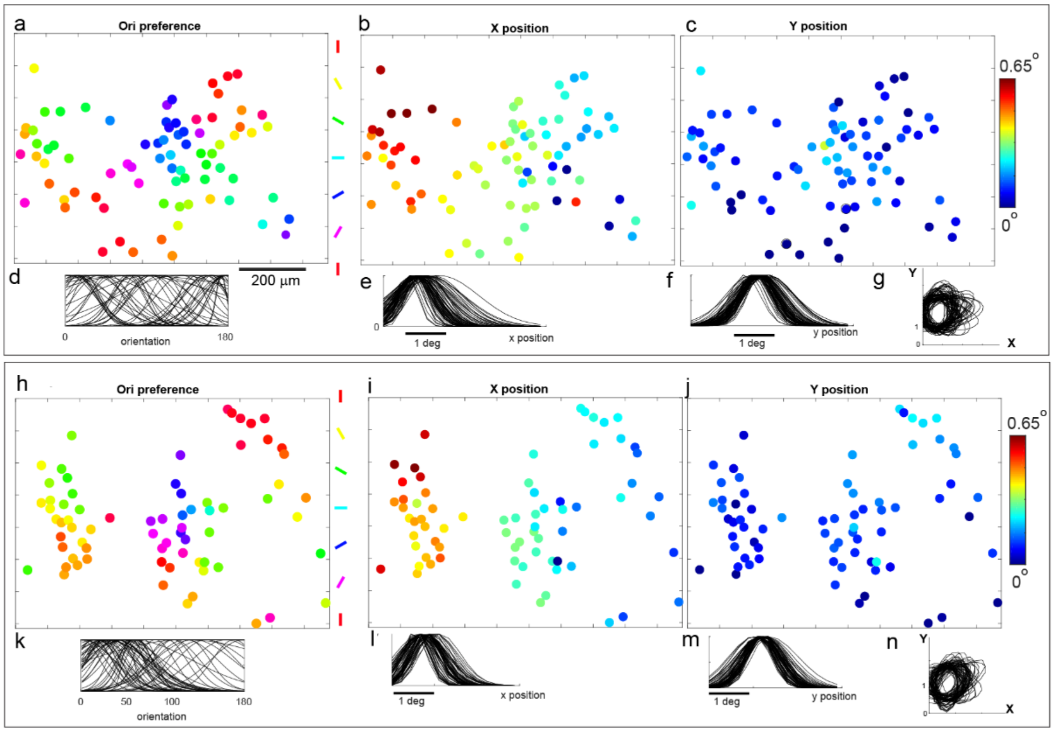

Figure S2: Measuring orientation tuning and RF envelopes from a random bar stimulus. The results of one ROI are depicted in the top panels (a-g), and the results of another ROI are in the bottom set of panels (h-n). They are the same ROIs as in Fig. S1. (a) Orientation preference map. It looks very similar to the map in 'S1a' obtained from the grating stimulus. (b) Map of horizontal, 'X', retinotopy. (c) Map of vertical, 'Y', retinotopy. (d) Overlay of all Gaussian fits to the orientation tuning curves from the ROI from 'a'. (e) Overlay of the x-axis projection of 2D Gaussian fits to the RF envelope. (f) Overlay of the y-axis projection of 2D Gaussian fits to the RF envelope. (g) 2D contour at 1σ wide of all the reconstructed RF envelopes in the ROI.

II. Logarithmic spatial frequency bandwidth as a function of f_o

The main text compared f_o against linear SF bandwidth (Fig. 5). Here, we extend this analysis to a comparison between f_o and logarithmic SF bandwidth, $\sigma_{\log(f)}$. Scale invariance predicts that $\sigma_{\log(f)}$ is independent of f_o (Eq. 3). Although the linear dependency between $\sigma_{\log(f)}$ and $\log_2(f_o)$ (Fig. S3a) is indeed shallow (slope = -0.31), the correlation is strong (r = -0.76; p=3.6e-34), which is consistent with previous reports (De Valois et al., 1982; Xing et al., 2004). To illustrate the consistency of this dependence with pooled scale invariance in the linear SF domain, we plugged the fit to linear bandwidth at the output of the pooling model (Eq. 7), $\sigma_{f,\mathrm{p}}(f_o)$, into Eq. 3,

$$\sigma_{\log(f)}(f_o) = \log_2[(f_o + \sigma_{f,\mathrm{p}}(f_o))/f_o] \qquad \text{Log SF bandwidth from pooled scale invariance (Eq. 8)}$$

which is the green curve in Figure S3a. These results provide a model to more formally describe the dependence of $\sigma_{\log(f)}$ on $\log_2(f_o)$, which is an invariant pooling parameter in the linear SF domain.

Next, we characterized the functional architecture of $\sigma_{\log(f)}$. The maps in Figure S3c are visibly much flatter than the f_o maps in Figure S1d,e, when placed in the same 5-octave color scale. However, strong clustering of $\sigma_{\log(f)}$ is expected given its dependence on f_o. Indeed, there is a significant local clustering of $\sigma_{\log(f)}$ (Fig. S3b, black) that follows the trend for f_o. The marginal and joint statistics of $\sigma_{\log(f)}$ and f_o are summarized in Table 1 and Table 2.

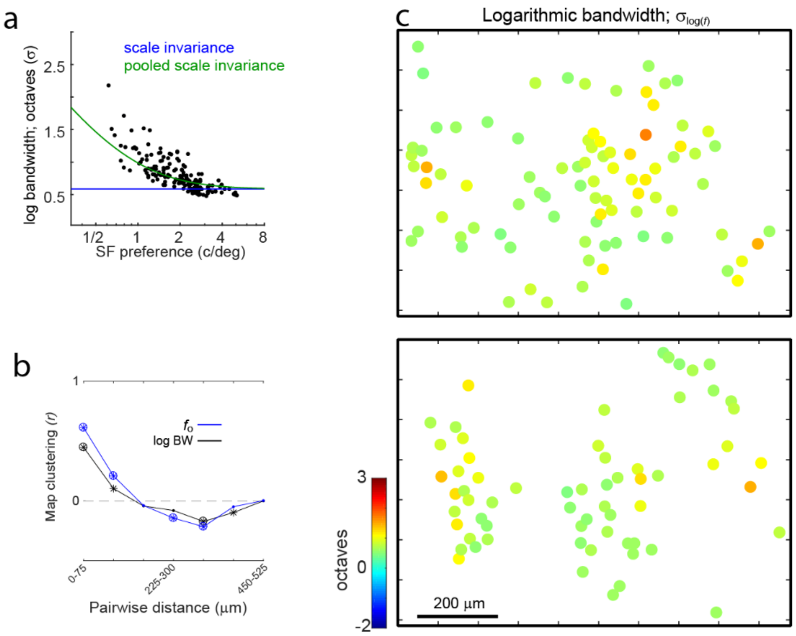

Figure S3: Characterizing the representation of f_o and logarithmic SF bandwidth ($\sigma_{\log(f)}$). (a) Scatter plot compares f_o (x-axis) to $\sigma_{\log(f)}$ (y-axis). Green line is the fit to the pooled scale invariance model for linear SF bandwidth (Eq. 7; Fig. 5a green), followed by a conversion to log SF bandwidth (Eq. 8). (b) Pearson correlation coefficient between pairs, at 75 μm cortical distance bins. Black and blue curves are clustering of $\sigma_{\log(f)}$ and f_o. Symbol at each distance bin indicates Pearson correlation level of significance: Small dot is $p>0.01$, asterisk is $p<0.01$, asterisk and open circle is $p<0.001$. (c) Maps of $\sigma_{\log(f)}$ at two depths and similar x/y location. Same ROIs as in Fig. S1.

III. More example tuning curves

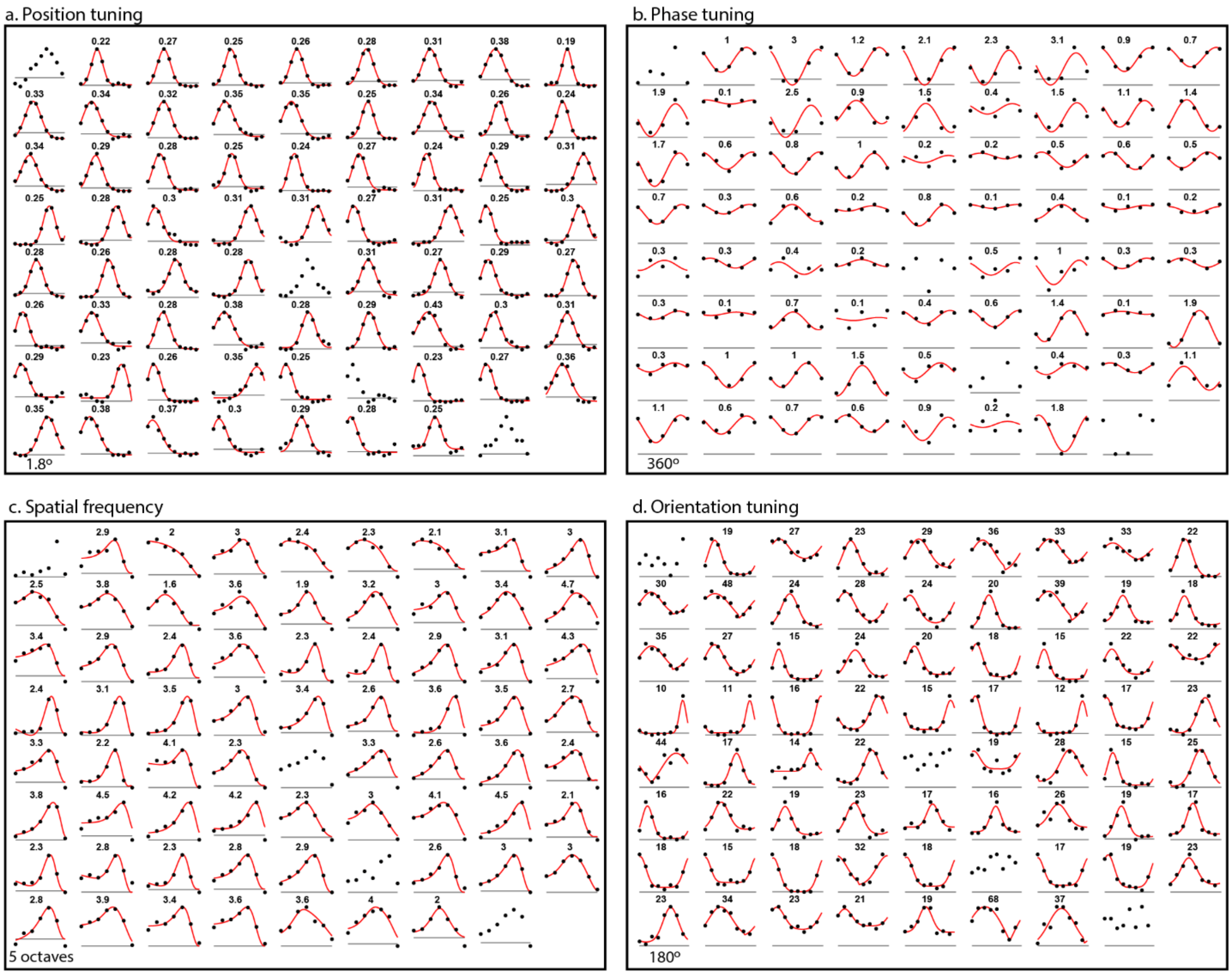

Figure S4: These panels show all of the tuning curves and fits from one of the three ROIs (viz. Fig. 7). Also, this population corresponds to the open circles in Figures 3a, 4a, 5a, 6a. The black dots are the raw data points and the red curves are the fits. 4 of the 71 neurons were excluded from analysis because of inaccurate fitting in one of the four response domains shown here. These excluded four do not show a red fit. The line under each tuning curve is at zero on the y-axis (gray). The text above each tuning curve is the selectivity metric. (a) Response as a function of bar position. The x-axis spans 1.8° of visual field. σ of Gaussian fit is shown in title. (b) Response to each phase of a grating, at the preferred orientation and SF. F1/F0 from sinewave fit is in each title. (c) Response to each SF (0.25 to 8 cyc/°). The difference-of-Gaussian fit gives the linear bandwidth (cyc/°) value in the title, as defined in Methods. (d) Response to each grating orientation. Title shows σ of Gaussian fit.

IV. Spike-to-fluorescence nonlinearities, and their potential effect on main results

It is well known that a nonlinearity resides within the transformation between spikes and the measured fluorescence of a calcium indicator (Yasuda et al., 2004), which can alter the measured width of tuning (Nauhaus et al., 2012b). Our use of reverse correlation stimuli can help to recover the tuning functions before they are passed through an output nonlinearity (Chichilnisky, 2001; Nauhaus et al., 2012b), yet this was not directly measured, so a more thorough characterization of how fluorescence nonlinearities may have affected our results is warranted. Here, we modeled the spike-to-fluorescence nonlinearity to infer its effect on our results. To begin, we consider a neuron's spike rate as a function of some arbitrary stimulus variable, 'x', which can be approximated by a Gaussian, $Aexp(-x^2/_{2\sigma^2})$. We then pass the Gaussian tuning curve through a power law nonlinearity to model the observed fluorescence tuning curve, $\left[Aexp(-x^2/_{2\sigma^2})\right]^{NL}$, where NL is a coefficient. The second moment of a Gaussian (or any distribution) scales inversely with NL, so the tuning at the output of the fluorescence nonlinearity has a new σ of

$\sigma/\sqrt{NL}$. This means that tuning will broaden for saturating nonlinearities (NL<1) and sharpen for accelerating nonlinearities (NL>1). Furthermore, the magnitude by which σ is scaled, $1/\sqrt{NL}$, is independent of the Gaussian's amplitude or width at the input of the nonlinearity. This means that scale invariance of RFs will be maintained if passed through a power law nonlinearity, in general. We make this derivation more concrete in the simulations described in sections IV.1 and IV.2 below. However, for nonlinearities in general, special circumstances may arise whereby they can introduce systematic biases in tuning that are not scale invariant. In section IV.3, we test for the possibility that deviations from scale invariance in our data are due to differing tuning amplitudes that are coupled to f_o.

IV.1 Can a fluorescence nonlinearity account for deviations from scale invariance?

A concern may be that the population in our data is scale invariant, yet a fluorescence nonlinearity makes it appear less so, and more consistent with the pooling model. However, the above derivation, and simulation in Figure S5, shows that scale invariance is maintained with a power law nonlinearity. Specifically, RF size and SF bandwidth would still scale in proportion to f_o, yet they have a different slope (Fig. S5a,b; dashed blue). Furthermore, the GCaMP6 nonlinearity is empirically accelerating (Chen et al., 2013), which sharpens the scale invariant tuning curves, pushing the scale invariant model down and further from the data. Applying a nonlinearity to a scale invariant population also cannot account for the trends between F1/F0 vs. f_o (Fig. S5c), or orientation bandwidth and f_o (Fig. S5d). To simulate F1/F0, post-nonlinearity, we squared a rectified sinewave, and then recomputed the F1/F0, which increases F1/F0 above π. In the case of orientation bandwidth, we again scaled the bandwidths by $1/\sqrt{NL}$, where NL = 2.

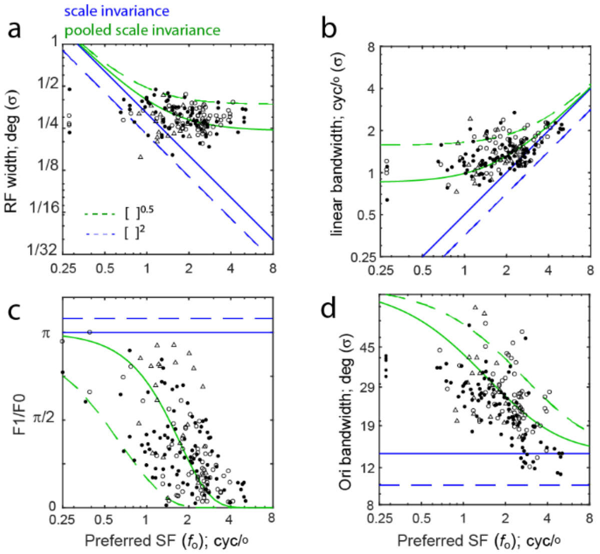

Figure S5: Modeling the effect of a fluorescence nonlinearity on main results. Each panel shows data (black) and models (solid blue and green lines) that were presented in the main text. Here, we also show the potential effect of a GCaMP6 nonlinearity on the measured tuning of a scale invariant population (dashed blue). We also asked how a GCaMP6 nonlinearity would affect the pooling model. (a) The same data and models are shown as in Figure 3a. The dashed blue line is the result of passing the scale invariant spatial tuning curves through an accelerating power law nonlinearity (e.g. GCaMP fluorescence); viz. squaring the tuning curves. The dashed green line is the result of re-fitting the pooling model after inverting the same accelerating nonlinearity; viz. applying $[\ ]^{0.5}$ to the measured tuning curves. (b,c,d) Same data and models are shown as in Figure 5a,4a,6a, respectively. The modeling of nonlinearities (dashed lines) was performed as in 'a'.

IV.2 How does a fluorescence nonlinearity effect the pooling model?

Although a power law nonlinearity cannot account for the deviation from scale invariance, it will affect the parameter values computed in the pooling model. Here, we assumed that the measured tuning curves were passed through a power law fluorescence nonlinearity, which allowed for a straightforward inversion of the nonlinearity to compute underlying tuning widths. In the case of an accelerating

nonlinearity with NL = 2, we would invert the nonlinearity by applying NL = 0.5, which entails scaling the measured σ by $1/\sqrt{0.5}$. This "correction" shifts the data points in Figs. S5a,b,d up by a factor of ~1.4. In the case of F1/F0, we passed the rectified sinewave through the same correction, and then recomputed the F1/F0. Raising the data points in this way increases their distance from the model of scale invariance (solid blue), which produces the new fit shown as dashed green in each panel. The pooling window to account for RF width is 0.24 deg (Fig S5a; solid green) and 0.37 deg (Fig S5a; solid green), before and after the inversion of the nonlinearity. Similarly, the pooling window in the SF domain changes from 0.85 cyc/° (Figs S5b,d, solid green) to 1.57 cyc/° (Figs S5b,d, dashed green). Other accelerating nonlinearites will change the pooling model in the same direction.

IV.3 Potential biases in response amplitude to the stimulus bar, as a function of SF tuning

In section IV.1 above, we showed that scale invariance is maintained after tuning curves are passed through a static nonlinearity. Also, note that scale invariance holds independent of the spike rates, when the nonlinearity can be approximated by a power law $[]^{NL}$. That is, the second moment of the tuning curve scales by 1/NL, regardless of the tuning curve's amplitude. However, other classes of nonlinearities are not scale invariant (Mineault et al., 2016). This is particularly relevant to the analysis of RF width vs. f_o (Fig. 3), which uses a 0.2° wide bar. As with any real stimulus, the bar's Fourier energy spectrum is not flat, so it is expected to drive some neurons more than others depending on their SF tuning. If a neuron's optimal bar width is a half cycle of f_o, then a 0.2° bar maximally drive neurons with f_o = 2.5 cyc/° – i.e. the bar width is suboptimal for any neuron with f_o above or below 2.5 cyc/°. Theoretically, this should not have a major impact on response amplitude, as 2.5 cyc/° lies inside the bandwidth of most neurons that we recorded – the median low cutoff (f_{low}) and high cutoff (f_{hi}) was 0.79 and 3.77 cyc/° (at 61% of peak), respectively. It should be noted that we chose a bar width that is optimized for the high side of this SF range to minimize the "smearing" in our RF measurements (section VI).

Next, we compared response amplitude to the bar stimulus against SF tuning. Again, a 0.2° bar maximally drives neurons with f_o = 2.5 cyc/°. We divided the study's population into 3 groups: 1) Neurons with f_{hi} < 2.5 cyc/° (the "low SF group" - 6%), 2) neurons with f_{low} > 2.5 cyc/° (the "high SF group" - 3%), and 3) neurons with f_{low} < 2.5 cyc/° > f_{hi} (the "optimal SF group" - 91%). We computed the mean response amplitude to the best bar for each neuron, and then the median of each of the 3 groups. The response amplitude of the high SF group was 23% lower than the optimal group, and the response amplitude of the low SF group was 5% lower than the optimal group. Lastly, we found no correlation between f_o and response amplitude to the bars (r = -0.17; p = 0.12). We conclude that most of the neurons are theoretically and empirically well driven by the 0.2-degree bars without significant dependence on their SF tuning, suggesting that nonlinearities – power law or otherwise - are unlikely to have a significant effect on RF width vs. f_o.

V. Performance comparison between pooled scale invariance and a descriptive linear model

In the Results section, "Summary of pooled scale invariance model and its performance", we showed that pooled scale invariance is dramatically superior to scale invariance at predicting the data. Here, we performed a similar analysis to compare pooled scale invariance against a descriptive linear model. We chose a line in log-log coordinates as the descriptive model given its simplicity and the linear appearance of the data, especially in the case of SF bandwidth (Fig. 5a) and orientation bandwidth (Fig. 6a). As noted previously, and as summarized in Table 2 (row 3), there is a significant correlation coefficient between log(f_o) and every other RF parameter measured. To further quantify relative performance between the linear model and pooled scale invariance, mean-squared error (MSE) distributions were computed for each of the following: $\log_2$(RF width), F1/F0, $\log_2$(SF bandwidth), and $\log_2$(orientation bandwidth). "Leave-one-out cross-validation" was used to create the prediction errors. Based on paired t-tests of MSE, the linear fit showed a subtle but significant improvement over scale invariant pooling model for $\log_2$(RF width) (p=0.004; 95% ci = [-0.06 -0.01]), $\log_2$(SF bandwidth) (p=0.006; 95% ci = [-0.04 -0.007]), $\log_2$(orientation bandwidth) (p=0.007; 95% ci = [-0.06 -0.01]), and F1/F0 (p=0.0004; 95% ci = [-5.1 -1.5]). At the same time, scale invariant pooling requires far fewer parameters (viz. 3) than fitting lines (viz. 8). Furthermore, unlike the linear fits, pooled scale invariance provides an intuitive amendment to the classic model of scale invariance. In conclusion, pooled scale invariance provides a more parsimonious description of the data, using parameters with meaningful units.

VI. Correcting for the effects of eye movements and stimulus bar width on RF size

Slow eye drift was measured by taking the average RF within roughly 4-minute blocks of data collection, in a 20-minute experiment. See title in each panel of Figure S6 for trial delineation. This gave 5 independent samples of relative eye position across the experiment. Although RFs from individual neurons are noisier when measured with so few trials, only the average RF position in the ROI is needed to estimate eye position, which will be far more accurate. Figure S6 shows the results from the ROI that yielded the strongest responses in our data set (Fig. S1, right panels), which allowed for the most accurate assessment of eye position from short blocks of time. This ROI gave RF widths that were representative of the entire data set (Fig. 3a, open circle data points). In summary, the X / Y range of movement was 0.104° / 0.059°, and the X / Y standard deviation across the 5 experimental windows was 0.039° / 0.025°. This is about 10x smaller than the RF widths (σ), so it's likely that some of the computed drift is due to inevitable measurement noise when computing RFs in a short time window. Nonetheless, we can use it to compute an upper limit of the effect of slow eye drift on the relationship between f_o and RF width reported in Figure 3.

The impact of eye movements on RF width can be modeled as an additional variable in Eq. 5, now giving $\sigma^2_{p'(x)} = \sigma^2_x(f_o) + \sigma^2_{h(x)} + \sigma_{EM}{}^2$, where $\sigma_{EM}{}^2$ is the variance of eye movements and $\sigma^2_{p'(x)}$ is the observed RF width that is inflated by the eye movements. The measured pooling window was $\sigma^2_{h(x)} = 0.24^2 = 0.058$, whereas the measured Y-axis variance in eye position was $\sigma_{EM}{}^2 = 0.039^2 = 0.0015$. We can then obtain a corrected estimate of $\sigma^2_{h(x)}$ by "deconvolving" the eye movements by subtracting the variance: $(0.24^2 - 0.039^2)^{0.5}$, or ~0.237. The corrected value of $\sigma_{h(x)}$ (0.237°) is only slightly different than the uncorrected value (0.24°). In conclusion, the magnitude of eye movements in the anesthetized, paralyzed experiment is unlikely to have an impact on the pooled scale invariance model in the spatial domain, which describes RF width and spatial phase selectivity as a function of f_o (Figs. 3&4).

Similar to eye drift, computing the effect of stimulus bar width (0.2°) on measured RF size can be performed by assuming the addition of variances through convolution. Here, the convolution is between a "rect" function (i.e. the bars) and a Gaussian (our 1D RF fits at the preferred orientation). A rect function has a second moment (i.e. variance) that is width^2/12. Convolution of the rect function with a Gaussian produces a Gaussian-like function that has a variance that is the sum of the rect and Gaussian variances. This may be provable analytically, but we instead confirmed this in simulation using Gaussian RFs and bar widths that were near the actual values. The variance of the bars is $\sigma_B{}^2 = 0.2^2/12$, so we can compute the corrected pooling window, $\sigma'_{h(x)}$, by deconvolving both bar width and eye movements by taking $\sigma'^2_{h(x)} = \sigma^2_{h(x)} - \sigma^2_{EM} - \sigma^2_B$ = $(0.24^2 - 0.039^2 - 0.058^2)^{0.5}$ ~ 0.23. In summary, there is little difference from the uncorrected estimate.

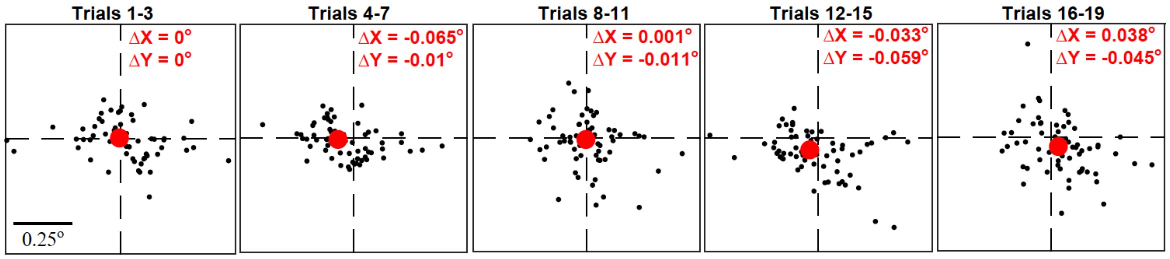

Figure S6: Measuring eye movements in 5 independent blocks of a 20 min experiment. Each panel shows the RF positions of all the RFs in the ROI (black dots), and the median RF is red. The positions are all relative to the mean RF position in the first block.

Next, although an analytical treatment of the smearing of the bar is given above, to help make this more concrete, we provide a simulation that compares the “actual” RF width against the “measured” RF width. This simulation closely mimics the measurement and analysis described in the Methods, such as the initial sample spacing in the stimulus, along with offline binning. We first simulated a population of scale invariant simple cells with ON and OFF subfields (Fig. S7a, solid lines). The relationship between RF width and f_o is consistent with the scale invariant model described in the main text. We then “measure” both ON and OFF subfields by convolving them with the 0.2° bar, then sample every 0.1°, then bin every two samples to obtain 0.2 °/sample (Fig. S7a, dashed lines). Next, we obtained the RF envelope by adding the subfields (Fig. S7b). Finally, as in Figure 3, the RF width is computed based on a Gaussian fit to the envelope. Figure S7c gives a summary of these simulated results, which has the same axes as Figure 3a. The effect of the stimulus and our analysis (black) cannot account for the deviation from scale invariance (green).

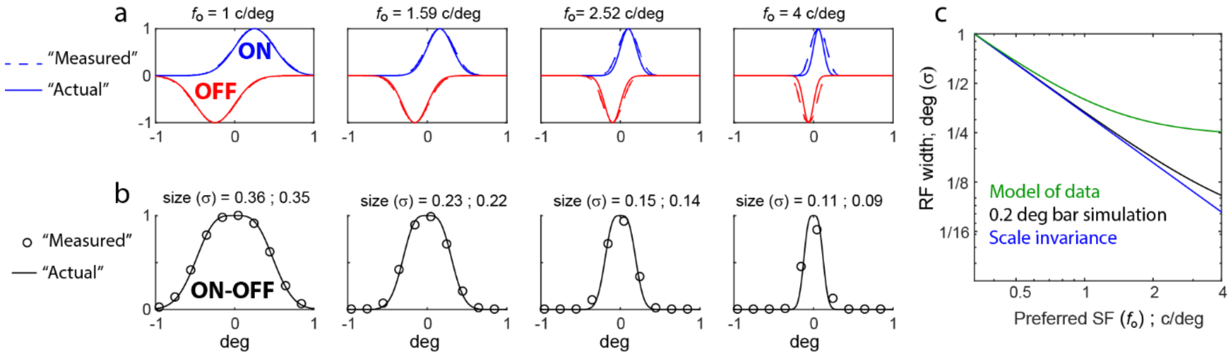

Figure S7: Simulating the effects of 0.2° bar stimuli on the measured RF width. (a) Each of the four panels shows the difference of Gaussian RF model for a different f_o. Solid line is the “actual” subfield. Dashed line is what can be measured with the 0.2° bars. i.e. it is the actual subfield convolved with the 0.2 ° bar. (b) The RF envelope; i.e. addition of the subfields shown in ‘a’. Solid line is the actual envelope. Dots simulate our measured data points of the RF envelope; they are the measured envelope sampled at 0.1°, followed by binning every two samples to achieve 0.2° sample rate. Title gives the RF width of the measured and actual RF width (degrees), respectively. (c) RF width vs. f_o plot, like Figure 3a in the manuscript. Specifically, the black curve comes from Gaussian fits to the simulated measurement of the envelope.

VII. Effects of neuropil subtraction on receptive field width

Here, we performed an analysis of neuropil subtraction to assess its impact on metrics of tuning selectivity. A tuning curve was computed for a region of pixels surrounding each neuron, and then subtracted from the tuning curve of the cell body in the middle. The widths of the “neuropil corrected” tuning curves were then re-computed exactly as described in the main text. We found that this procedure had very little effect on RF width, SF bandwidth, and orientation bandwidth.

Like prior studies (Bonin et al., 2011; Kerlin et al., 2010), we assume that the measured tuning curve from each cell body ($\boldsymbol{K}_{\text{cellBody}}$) is a weighted sum of two tuning curves: one from the neuron itself ($\boldsymbol{K}_{\text{neuron}}$) and one from the surrounding neuropil ($\boldsymbol{K}_{\text{neuropil}}$).

$$\boldsymbol{K}_{\text{cellBody}} = \boldsymbol{K}_{\text{neuron}} + \alpha \boldsymbol{K}_{\text{neuropil}} \qquad \textit{Supp. Eq. 1}$$

To compute the neuropil signal, $\boldsymbol{K}_{\text{neuropil}}$, we took a Gaussian-weighted average of pixels surrounding each cell body, where the 2D Gaussian σ = 8 μm. The Gaussian-weighted average excluded pixels in the cell body ROI.

We asked the following: What happens to tuning width as one subtracts variable amounts of $\boldsymbol{K}_{\text{neuropil}}$? More specifically, how does the tuning width computed from $\widehat{\boldsymbol{K}}_{\text{neuron}} = \boldsymbol{K}_{\text{cellBody}} - \alpha \boldsymbol{K}_{\text{neuropil}}$ change as a function of α? We note that subtracting neuropil tuning from cell body tuning is equivalent to subtracting the timecourse of the neuropil from the timecourse of the cell body, followed by computing the subsequent tuning. This equivalence is due to the fact that computing each tuning curve is a linear operation.

For simplicity, we simply discuss this analysis in the context of the former operation, the subtraction of neuropil tuning from cell body tuning.

To begin the analysis, we first normalized the amplitude of our two known tuning curves, $\boldsymbol{K}_{\mathrm{cellBody}}$ and $\boldsymbol{K}_{\mathrm{neuropil}}$, to each have a peak of 1. Next, $\widehat{\boldsymbol{K}}_{\mathrm{neuron}}(\alpha,i) = \boldsymbol{K}_{\mathrm{cellBody}}(i) - \alpha\boldsymbol{K}_{\mathrm{neuropil}}(i)$ was computed for each cell body (i) and a range of coefficient values; $\alpha = [0\ .2\ .4\ .6\ .8]$. Next, a function was fit to each $\widehat{\boldsymbol{K}}_{\mathrm{neuron}}(\alpha,i)$ in the manner described in the Methods. This general procedure was performed for RF width, SF bandwidth, and orientation bandwidth (Fig. S8). This gave a tuning width for each cell, as a function of α. As α increased, $\widehat{\boldsymbol{K}}_{\mathrm{neuron}}(\alpha,i)$ from most cells began to look less like a Gaussian and therefore less likely to yield an accurate tuning fit, which was quantified with % variance accounted for by the fit. A plot of %variance vs. α is shown in Figures S8a-c. In each case, there is a steady drop in the average goodness-of-fit.

Figure S8d plots the mean and SE of the RF width as a function of the amount of neuropil subtraction, α. For each neuron, the RF width at each level of α was normalized by the RF width without any neuropil subtraction. Specifically, the error bars show the mean and SE of $\sigma(\alpha,i)/\sigma(0,i)$, across all three imaging regions. Note that $\sigma(0,i)$ is how widths were calculated in the main text. There is a shallow drop in RF width as α increases to 0.6. At $\alpha = 0.6$, the RF widths shrank by approximately 7%. In the case of SF bandwidth, there was not a noticeable change with neuropil subtraction. In the case of orientation bandwidth, it shrank by 18% when $\alpha = 0.8$. The relative narrowing of orientation bandwidth by this procedure is likely due to more heterogeneity in the neighboring preferred orientation map, relative to the retinotopy or f_o map. We did not show the case of $\alpha = 1.0$, as this reduces the amplitude of the tuning curves to zero.

In summary, we calculated changes in tuning width, from modest to extreme levels of neuropil subtraction. Neither end of the spectrum supports the notion that neuropil significantly broadened the tuning measurements in the main Results. An alternative scenario is that the signals at cell bodies are completely swamped by the neuropil. However, this seems extremely unlikely based on previous studies with GCaMP6, along with the fact that cell body ROIs were identified as regions with the highest independence from the surround (Fig 2b).

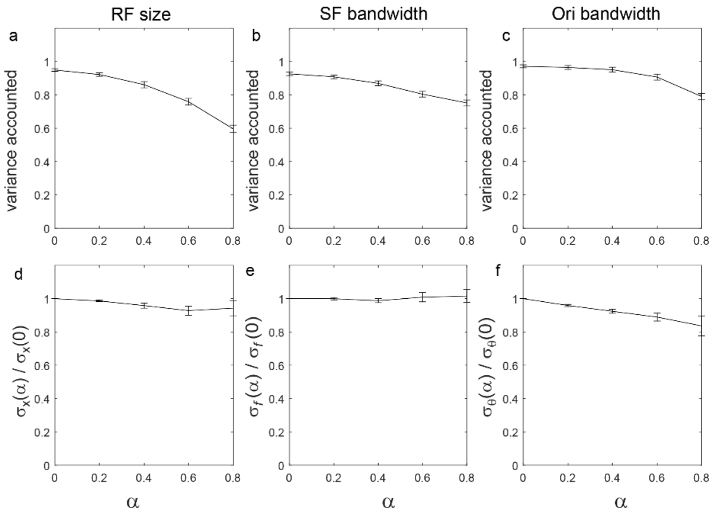

Figure S8: Effects of neuropil subtraction on tuning width. The error bars in ‘a’ and ‘d’ show the mean and SE across the same population of 157 neurons as in Figure 3a (2 animals, 3 ROIs). The error bars in ‘b’,’c’,’e’, and ’f’ show the mean and SE across the same population of 174 neurons as in Figures 5a,6a (2 animals, 3 ROIs). (a) Dependence of the quality of a Gaussian fit (spatial envelope) on the magnitude of neuropil subtraction. X-axis is the amount of neuropil subtraction; i.e. α in Supp. Eq. 1. Y-axis is the %variance accounted for by the fit to the RF along the axis of optimal orientation. (b) Same as ‘a’, but for SF bandwidth. (c) Same as ‘a’, but for orientation bandwidth. (d) Dependence of RF width on the magnitude of neuropil subtraction. X-axis is the amount of neuropil subtraction; i.e. α in Supp. Eq. 1. Y-axis is the ratio of RF width (after neuropil subtraction), over the RF width (before neuropil subtraction). Only values out to α = 0.8 are shown because values greater than this are difficult to interpret due to poor Gaussian fits. (e) Same as ‘d’, but for SF bandwidth. (f) Same as ‘d’, but for orientation bandwidth.

VIII. Recording chamber

One of the two animals was initially used for widefield recording in the awake preparation, and had an imaging chamber as described in (Seidemann et al., 2016). In this case, the widefield chamber was removed and replaced with a recording chamber and window used for an acute imaging session, as described in (Nauhaus et al., 2012a). The second animal was initially implanted with a chamber designed for chronic two-photon imaging, illustrated in Figure S9.

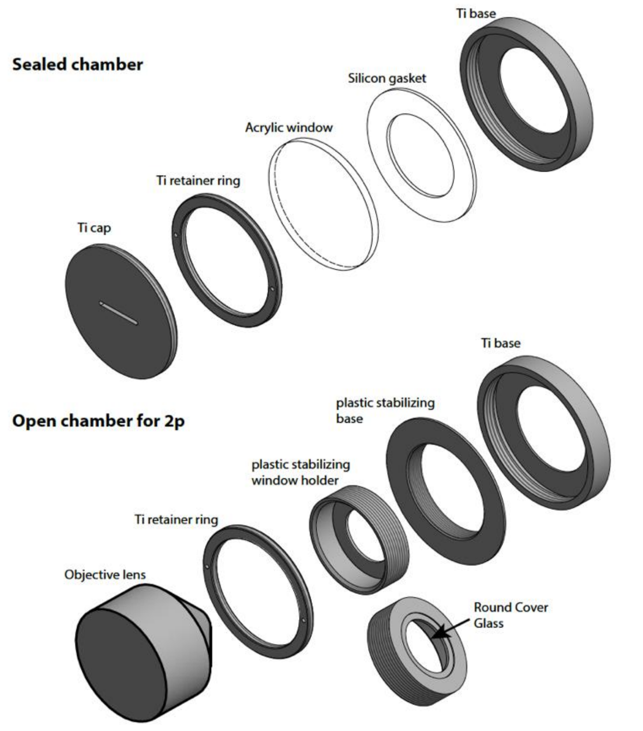

Figure S9: Imaging chamber in the "Sealed" and "Open" configuration. "Sealed chamber": The titanium base at the top right is the one chronically implanted element. The inner diameter is 24 mm, the inner threading is M36, and the total height is 5.7mm. The next elements are layered to form a sealed chamber that can be viewed by removing the top cap. "Open chamber for 2p": For two-photon imaging the "stabilizing window holder" is screwed into the "stabilizing base" by an amount that keeps the brain stable. Multiple versions of these plastic elements were machined in order to accommodate variable brain position and desired imaging location. One version of the window holder had M18 threading, a 10 mm aperture, and held a 12 mm diameter cover glass.

References

Akerboom, J., Chen, T.-W., Wardill, T.J., Tian, L., Marvin, J.S., Mutlu, S., Calderón, N.C., Esposti, F., Borghuis, B.G., Sun, X.R., et al. (2012). Optimization of a GCaMP calcium indicator for neural activity imaging. J. Neurosci. Off. J. Soc. Neurosci. *32*, 13819–13840.

Bonin, V., Histed, M.H., Yurgenson, S., and Reid, R.C. (2011). Local diversity and fine-scale organization of receptive fields in mouse visual cortex. J. Neurosci. Off. J. Soc. Neurosci. *31*, 18506–18521.

Chen, T.-W., Wardill, T.J., Sun, Y., Pulver, S.R., Renninger, S.L., Baohan, A., Schreiter, E.R., Kerr, R.A., Orger, M.B., Jayaraman, V., et al. (2013). Ultrasensitive fluorescent proteins for imaging neuronal activity. Nature *499*, 295–300.

Chichilnisky, E.J. (2001). A simple white noise analysis of neuronal light responses. Netw. Bristol Engl. *12*, 199–213.

De Valois, R.L., Albrecht, D.G., and Thorell, L.G. (1982). Spatial frequency selectivity of cells in macaque visual cortex. Vision Res. *22*, 545–559.

DeAngelis, G.C., Ghose, G.M., Ohzawa, I., and Freeman, R.D. (1999). Functional micro-organization of primary visual cortex: receptive field analysis of nearby neurons. J. Neurosci. Off. J. Soc. Neurosci. *19*, 4046–4064.

Kerlin, A.M., Andermann, M.L., Berezovskii, V.K., and Reid, R.C. (2010). Broadly tuned response properties of diverse inhibitory neuron subtypes in mouse visual cortex. Neuron *67*, 858–871.

Mineault, P.J., Tring, E., Trachtenberg, J.T., and Ringach, D.L. (2016). Enhanced Spatial Resolution During Locomotion and Heightened Attention in Mouse Primary Visual Cortex. J. Neurosci. Off. J. Soc. Neurosci. *36*, 6382–6392.

Nauhaus, I., Nielsen, K.J., Disney, A.A., and Callaway, E.M. (2012a). Orthogonal micro-organization of orientation and spatial frequency in primate primary visual cortex. Nat. Neurosci. *15*, 1683–1690.

Nauhaus, I., Nielsen, K.J., and Callaway, E.M. (2012b). Nonlinearity of two-photon Ca2+ imaging yields distorted measurements of tuning for V1 neuronal populations. J. Neurophysiol. *107*, 923–936.

Nauhaus, I., Nielsen, K.J., and Callaway, E.M. (2016). Efficient Receptive Field Tiling in Primate V1. Neuron *91*, 893–904.

Seidemann, E., Chen, Y., Bai, Y., Chen, S.C., Mehta, P., Kajs, B.L., Geisler, W.S., and Zemelman, B.V. (2016). Calcium imaging with genetically encoded indicators in behaving primates. ELife *5*.

Xing, D., Ringach, D.L., Shapley, R., and Hawken, M.J. (2004). Correlation of local and global orientation and spatial frequency tuning in macaque V1. J. Physiol. *557*, 923–933.

Yasuda, R., Nimchinsky, E.A., Scheuss, V., Pologruto, T.A., Oertner, T.G., Sabatini, B.L., and Svoboda, K. (2004). Imaging calcium concentration dynamics in small neuronal compartments. Sci. STKE Signal Transduct. Knowl. Environ. *2004*, pl5.
