# Supplementary material for: Uniform spatial pooling explains topographic organization and deviation from receptive-field scale invariance in primate V1
Source: Nat Commun. 2020 Dec 15;11:6390. doi: 10.1038/s41467-020-19954-9 (PMC7738493; doi:10.1038/s41467-020-19954-9)
Supplement: Supplementary file 3 — Reporting Summary [file 41467_2020_19954_MOESM3_ESM.pdf]

nature research

Corresponding author(s): Ian Nauhaus

Last updated by author(s): Oct 24, 2020

Reporting Summary

Nature Research wishes to improve the reproducibility of the work that we publish. This form provides structure for consistency and transparency in reporting. For further information on Nature Research policies, seeAuthors & Referees and theEditorial Policy Checklist .

Statistics

For all statistical analyses, confirm that the following items are present in the figure legend, table legend, main text, or Methods section.

n/a	Confirmed	
☐	☒	The exact sample size (*n*) for each experimental group/condition, given as a discrete number and unit of measurement
☒	☐	A statement on whether measurements were taken from distinct samples or whether the same sample was measured repeatedly
☐	☒	The statistical test(s) used AND whether they are one- or two-sided<br>*Only common tests should be described solely by name; describe more complex techniques in the Methods section.*
☐	☒	A description of all covariates tested
☒	☐	A description of any assumptions or corrections, such as tests of normality and adjustment for multiple comparisons
☐	☒	A full description of the statistical parameters including central tendency (e.g. means) or other basic estimates (e.g. regression coefficient) AND variation (e.g. standard deviation) or associated estimates of uncertainty (e.g. confidence intervals)
☐	☒	For null hypothesis testing, the test statistic (e.g. *F*, *t*, *r*) with confidence intervals, effect sizes, degrees of freedom and *P* value noted<br>*Give P values as exact values whenever suitable.*
☒	☐	For Bayesian analysis, information on the choice of priors and Markov chain Monte Carlo settings
☒	☐	For hierarchical and complex designs, identification of the appropriate level for tests and full reporting of outcomes
☐	☒	Estimates of effect sizes (e.g. Cohen's *d*, Pearson's *r*), indicating how they were calculated

Our web collection on statistics for biologists contains articles on many of the points above.

Software and code

Policy information about availability of computer code

Data collection	All visual stimuli were generated using custom routines that depended on MatLab 2016b, and Psychtoolbox v3.0.12. Two-photon data was collected using Scanbox software (no version given).
Data analysis	Data was analyzed using custom code in Matlab 2016b. In addition, movement correction software was used from Scanbox (no version given).

For manuscripts utilizing custom algorithms or software that are central to the research but not yet described in published literature, software must be made available to editors/reviewers. We strongly encourage code deposition in a community repository (e.g. GitHub). See the Nature Research guidelines for submitting code & software for further information.

Data

Policy information about availability of data

All manuscripts must include a data availability statement. This statement should provide the following information, where applicable:

- Accession codes, unique identifiers, or web links for publicly available datasets
- A list of figures that have associated raw data
- A description of any restrictions on data availability

The data from this study is available from the corresponding author upon reasonable request.

Field-specific reporting

Please select the one below that is the best fit for your research. If you are not sure, read the appropriate sections before making your selection.

☒ Life sciences ☐ Behavioural & social sciences ☐ Ecological, evolutionary & environmental sciences

Life sciences study design

All studies must disclose on these points even when the disclosure is negative.

Sample size	No statistical measures were used to determine the correct sample size. However, sample sizes are similar to those used in other primate studies.
Data exclusions	20% of neurons were excluded based on the variance accounted for by model fits. This excluded neurons with inaccurate parameterization and subsequent quantification.
Replication	Results were successfully replicated in three regions-of-interest.
Randomization	Participants were not allocated into groups. No subject randomization was implemented.
Blinding	Blinding is not relevant. Two macaque monkeys were used. There were no differences between the subjects that would conceivably create biases.

Reporting for specific materials, systems and methods

We require information from authors about some types of materials, experimental systems and methods used in many studies. Here, indicate whether each material, system or method listed is relevant to your study. If you are not sure if a list item applies to your research, read the appropriate section before selecting a response.

Materials & experimental systems

n/a	Involved in the study	
☒	☐	Antibodies
☒	☐	Eukaryotic cell lines
☒	☐	Palaeontology
☐	☒	Animals and other organisms
☒	☐	Human research participants
☒	☐	Clinical data

Methods

n/a	Involved in the study	
☒	☐	ChIP-seq
☒	☐	Flow cytometry
☒	☐	MRI-based neuroimaging

Animals and other organisms

Policy information about studies involving animals; ARRIVE guidelines recommended for reporting animal research

Laboratory animals	We used two adult male rhesus monkeys (Macaca mulata), ages 6 and 13.
Wild animals	They were obtained from a breeding facility. No wild animals were used.
Field-collected samples	No field collected samples were used in this study.
Ethics oversight	All procedures were conducted in accordance with guidelines of the US National Institutes of Health and were approved by the Institutional Animal Care and Use Committee (IACUC) at the University of Texas at Austin, which maintains accreditation from the American Association for Accreditation of Laboratory Animal Care (AAALAC).

Note that full information on the approval of the study protocol must also be provided in the manuscript.
